# Regorafenib-related erythrocytosis in metastatic extra-gastrointestinal stromal tumor: a case report

**DOI:** 10.3389/fonc.2024.1398055

**Published:** 2024-08-06

**Authors:** Elena Fassi, Vito Amoroso, Deborah Cosentini, Vittorio Ferrari, Marta Laganà, Alfredo Berruti, Pierluigi di Mauro

**Affiliations:** Medical Oncology Unit, Department of Medical and Surgical Specialties, Radiological Sciences, and Public Health, University of Brescia. ASST Spedali Civili, Brescia, Italy

**Keywords:** erythrocytosis, regorafenib, polycythemia, EGIST, TKIs

## Abstract

**Introduction:**

Regorafenib is an oral multi-targeted tyrosine kinase inhibitor (TKI) indicated for the treatment of various tumor types, including metastatic gastrointestinal stromal tumors (GIST), as a third-line systemic therapy. Erythrocytosis, which is characterized by an increase in erythrocyte count, hemoglobin, and hematocrit levels, has been described as a side effect of some antiangiogenic TKIs but has never been associated with regorafenib administration.

**Case presentation:**

An extra-GIST was diagnosed in a 58-year-old woman after she underwent surgery to remove a pelvic mass. Three years later, systemic therapy with imatinib was started due to pelvic disease recurrence. However, after six months, due to disease progression, we prescribed sunitinib, which the patient received for four years. Regorafenib was initiated in June 2019, and after six months, we noted an increase in the erythrocytes’ count and hemoglobin (Hb) levels. Given that the patient had clinical benefit and hematocrit was within normal range, we only monitored the blood cell count and continued to give regorafenib at the same dose. The drug was then stopped for over six weeks due to hospitalization for severe acute respiratory syndrome coronavirus 2 (SARS-CoV-2) infection, and Hb levels returned to normal. Therefore, we decided to restart regorafenib at a lower dose. However, Hb levels rose again in conjunction with increased hematocrit, resulting in the need for multiple phlebotomies. We attempted to restart regorafenib every other day, but it was unsuccessful, so we stopped it permanently in May 2023, and all values returned to normal.

**Conclusion:**

Regorafenib may cause secondary erythrocytosis that could not be dose-related, as this case report suggests. Secondary erythrocytosis might be a marker of TKI efficacy, given the patient’s prolonged clinical benefit during regorafenib treatment (48 months). In patients receiving regorafenib, monitoring blood count as well as any symptoms associated with erythrocytosis may be suggested.

## 1 Introduction

Gastrointestinal stromal tumors (GISTs) are the most common mesenchymal tumors of the gastrointestinal tract (1, 2). They probably arise from interstitial Cajal (ICC) cells, which regulate gastrointestinal motility and, in most cases, have oncogenic gain-of-function mutations in KIT or PDGFRA genes (3). Extra-gastrointestinal stromal tumors (EGISTs) are a rare subtype that originate outside the gastrointestinal tract, mainly from the mesentery, omentum, or peritoneum (4). Their incidence is estimated to be between 5% and 10% of all GISTs (5). Given the rarity of EGISTs, recommended treatment approaches are not different compared to GISTs. In particular, surgery is the first treatment choice for localized disease, while systemic therapy with specific tyrosine kinase inhibitors (TKIs) is indicated in metastatic or unresectable disease (6). Patients with metastatic disease characterized by KIT mutations receive imatinib as first-line therapy. In the case of disease progression, the standard second-line treatment for GIST is sunitinib, while regorafenib is approved as the third-line (2, 6).

Regorafenib is an oral multi-targeted TKI, whose targets are involved in the regulation of tumor angiogenesis (VEGFR1–3 and TEK), oncogenesis (KIT, RET, RAF1, BRAF, and and BRAF^V600E^), and maintenance of the tumor microenvironment (PDGFRA, PDGFRB and FGFR) (7). Fatigue, diarrhea, hand-foot skin reaction, and hypertension are the most commonly reported adverse events with this drug (8–10).

Erythrocytosis is defined as an increase in erythrocyte count, hemoglobin (Hb), and hematocrit (Hct) levels beyond the sex-specific normal range (11). Primary erythrocytosis is secondary to myeloproliferative diseases such as polycythemia vera (PV), while secondary erythrocytosis is associated with elevated serum erythropoietin (EPO) levels (11). This increase could be determined by hypoxia, solid tumors that produce EPO, congenital causes (e.g., erythropoietin receptor mutations or increased Hb-oxygen affinity), or drugs (12). Specific World Health Organization (WHO) criteria should be satisfied for diagnosing PV (13), while clinical and laboratory testing is necessary to diagnose secondary erythrocytosis. In particular, patients should undergo a drug history and a complete cardio-pulmonary evaluation to identify any signs of hypoxia, carbon monoxide exposure, or obstructive sleep apnea. Additionally, abdominal imaging examination may identify erythropoietin-producing tumors (11).

Here, we report a case of regorafenib-related secondary erythrocytosis in a patient treated for metastatic EGIST.

## 2 Case presentation

In 2011, a 58-year-old patient underwent surgical removal of a pelvic mass (16x10x15cm) and hysterectomy with bilateral salpingo-oophorectomy. The histological examination revealed a morphologically biphasic mesenchymal neoplasm with spindle and epithelioid cells; the mitotic index was less than 5/50 high-power field (HPF) and the Ki-67 labelling index was 5%. The tumor cells tested positive for CD117, P16, and SMA but negative for CD34 (**Table 1**). Based on the morphological features and the knowledge that CD34 is negative in 50% of EGIST cases (14, 15), a low-to-moderate EGIST was diagnosed. The assessment of KIT and PDGFRA mutations was not performed because it was not required at the time of the first diagnosis. Preoperative staging with CT scan did not show other lesions. According to the pathological stage (pT4N0M0) and high risk of recurrence based on Miettinen’s criteria (16), the treating physician could have recommended adjuvant therapy with imatinib; however, the patient only started a clinical and imaging-based follow-up program.

**Table 1:** Immunohistochemical analysis performed on primary tuomor at baseline.

	Positive	Negative
CD117	x	
SMA	x	
P16	x	
DOG1	x	
CD34		x
CAM 5,2		x
CK34betaE12		x
Cromogranin		x
HMB45		x
Inibin		x
Calretinin		x
S100		x
Melan A		x
Desmin		x
CD21		x
CD23		x
ER		x
EMA		x

**Figure 1:**
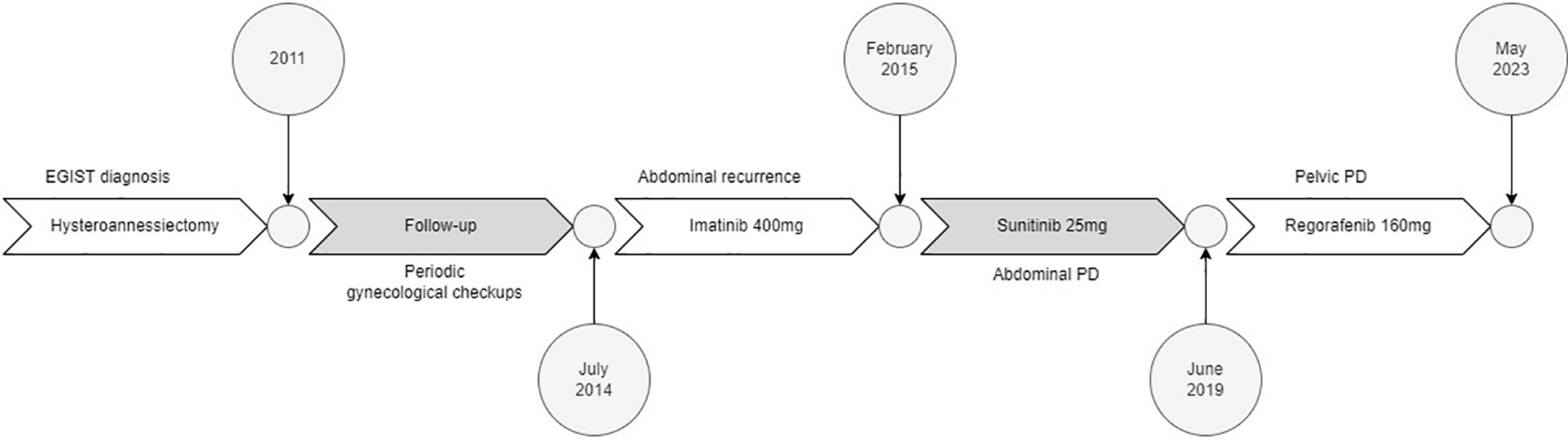
Drugs’ timeline.

**Figure 2:**
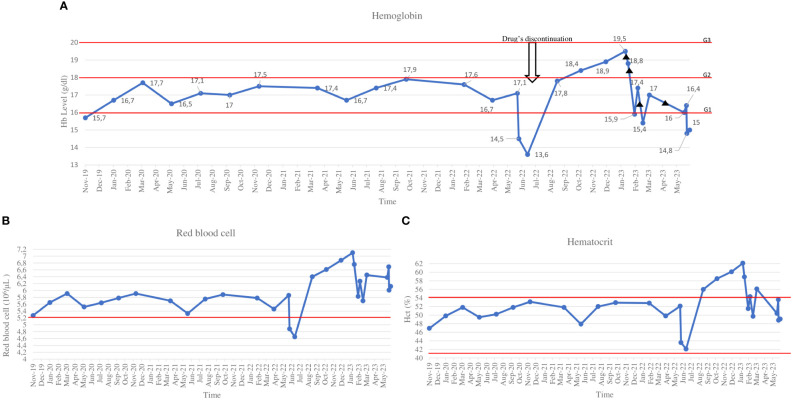
Hemoglobin **(A)**, red blood cells **(B)** and hematocrit **(C)** trend. ▲ phlebotomy.

Three years later, a gynecologic ultrasound examination revealed three pelvic non-cystic lesions (38x40x33mm, 78x42x54mm, and 27x17mm), which were confirmed as malignant relapse with an abdominal computed tomography. Then, the patient was referred to our Institution and started first-line treatment with imatinib 400 mg/day, which was associated with recurrent neutropenia of grade 2 (G2). After six months of therapy, peritoneal disease progression was documented after a radiological evaluation; therefore, second-line treatment with sunitinib continuous daily dosing of 25 mg was prescribed at a lower dose because of previous neutropenia secondary to imatinib. The treatment was well tolerated except for recurrent G2 neutropenia, after which the sunitinib dose was reduced to 12,5 mg/day. After four years of sunitinib therapy, further peritoneal disease progression was noted, and regorafenib 160 mg/day (once daily for 3 weeks followed by 1 week off-therapy) was prescribed as a third-line treatment in June 2019 (**Figure 1**). Six months after starting regorafenib, an increase in Hb level to 16.7 g/dl (normal range, 12-16 g/dl), which correspond to G1 according to CTCAE v 5.0, and in erythrocyte count to 5.65x10^6^/μL (normal range, 4.0-5.2x10^6^/μL) was detected during routine blood examinations. In the following months, the Hb level continued to slightly rise to 17.6 g/dl (G1) without symptoms onset or significant increase in Hct values: considering the clinical benefit the same dose of regorafenib was maintained. However, following the patient’s hospitalization due to severe acute respiratory syndrome coronavirus 2 (SARS-CoV-2) infection in June 2022, regorafenib was discontinued for over six weeks and the Hb levels decreased to normal values (13.6 g/dl at hospital discharge). Considering the patient’s ongoing clinical frailty after hospitalization and previous oncological treatments, as well as the prolonged disease stability, regorafenib was resumed at a lower dose (40 mg/day). Despite dose reduction, Hb levels rapidly increased to a maximum value of 19.5 g/dl (G2), with 60% of Hct value (normal range 41-54%) in January 2023. EPO level was 21.4 mU/ml (normal range 2.59-18.50 mU/ml). The patient remained asymptomatic. After immediately stopping regorafenib and hematologic consultation, the patient underwent phlebotomies, resulting in normalized Hb and Hct levels after two sessions. We attempted to restart the TKI therapy at the same dose, but a third phlebotomy session was necessary after one week, as the Hb level rose to 17.4 g/dl (G1) and the Hct was 54.3%. Regorafenib was restarted at 40 mg every other days as a final attempt; however, the patient required another phlebotomy after two weeks, so we ultimately stopped TKI therapy in May 2023 (time to treatment failure after regorafenib initiation: 48 months). Afterwards, the Hb and Hct levels finally returned to normal values. (**Figure 2**).

The patient remained asymptomatic, and the diagnostic testrequested by the hematologist demonstrated no JAK V617F mutation, thereby excluding the possibility of primary erythrocytosis.

The patient did not take any other concomitant medications throughout the entire period.

CT re-evaluation revealed pelvic disease stability. Since the patient had a good performance status, we tried a re-challenge treatment with imatinib 200mg/day in August 2023. Unfortunately, in December 2023, the patient developed a systemic infection that led her to death. The patient’s overall survival from the first diagnosis was 151 months (12 years and seven months).

## 3 Discussion

To our knowledge, we described the first case of regorafenib-related erythrocytosis in a patient diagnosed with metastatic EGIST who achieved a prolonged clinical benefit with third-line regorafenib therapy. We assessed the probability that erythrocytosis was a regorafenib-related side effect using the Naranjo scale (17, 18), obtaining a score of 6, corresponding to a probable correlation. Furthermore, no other EGIST cases with erythrocytosis are described in the literature. Nevertheless, in a preclinical study, mice with a particular loss-of-function mutation in KIT developed polycythemia vera in addition to GIST, suggesting a function of KIT in early erythropoiesis (19).

In patients with different tumor types, elevated Hb levels have been documented in association with multiple TKIs, including sunitinib (20), sorafenib (21), axitinib (22), and pazopanib (23).

While the exact mechanism of TKI-related erythropoiesis is still unknown, different hypotheses have been proposed. In a preclinical study, Tam et al. showed that endogenous VEGF plays a role in the down-regulation of vertebrate erythropoiesis (24). They also demonstrated that complete inhibition of VEGF can induce erythrocytosis and hepatic EPO synthesis (24). Van Der Veldt et al. hypothesized that sunitinib-related erythrocytosis may be secondary to vasoconstriction caused by VEGFR-2 inhibition that leads to intravascular fluid leakage. The decrease in plasma volume results in a relative increase in Hb, Hct, and erythrocytes (20). Alexandrescu et al. proposed that erythrocytosis could result from sensitization to the effects of EPO and is a rebound to VEGF inhibition-induced hypoxia after observing reversible erythrocytosis in patients treated with sunitinib or sorafenib for different tumor types (renal cell carcinoma [RCC], melanoma, and hepatocellular carcinoma) (21). Wang et al. reported a case series of six metastatic RCC patients treated with various anti-VEGF drugs (but not regorafenib) who developed polycythemia (25). They concluded that polycythemia was secondary to the release of EPO by malignant clones in response to anti-VEGF therapy, considering that RCC cells can synthesize EPO (26). Tripathi et al. conducted a retrospective analysis of 71 patients with advanced clear-cell RCC treated with different anti-VEGFR TKIs. They found Hb elevation in 83% of them, with a median time to increase of 2.4 weeks (27). However, higher Hb levels were associated with poor prognosis in this study. They assumed that hypoxia caused by anti-VEGF drugs led to the activation of other pro-angiogenic pathways that resulted in treatment resistance (27).

Similarly to other anti-VEGFR TKIs, we hypothesized that regorafenib could induce secondary erythrocytosis, even if the mechanism is unclear. Our patient continued to develop erythrocytosis even after we attempted to restart the drug at a reduced dose, suggesting that this adverse effect was not dose-related. In addition, considering that Hb levels increased six months after regorafenib initiation, we cannot exclude a potential sensitization effect.

Furthermore, in our patient, the clinical benefit was significantly prolonged with regorafenib therapy compared to what was reported in clinical trials (47 months vs 4.8 months) (9), despite EGISTs are characterized by a lower survival in comparison to other GISTs. Secondary erythrocytosis may result from VEGFR inhibition (25). Hence, the development of erythrocytosis could be a potential marker of regorafenib efficacy based on the prolonged response highlighted in this case. However, these observations are only hypothesis-generating and should be validated by further empirical research. Therefore, close monitoring of Hb, Hct, and erythrocytes levels, as well as any symptoms associated with erythrocytosis may be suggested in patients treated with regorafenib and exploring the potential association between the occurrence of erythrocytosis and the clinical outcome might be warranted.

## Data availability statement

The original contributions presented in the study are included in the article/supplementary material. Further inquiries can be directed to the corresponding author.

## Ethics statement

Written informed consent was obtained from the individual(s) for the publication of any potentially identifiable images or data included in this article.

## Author contributions

EF: Writing – original draft, Writing – review & editing, Conceptualization. VA: Methodology, Supervision, Writing – review & editing. DC: Writing – review & editing. VF: Supervision, Writing – review & editing, Conceptualization. ML: Writing – review & editing. AB: Supervision, Writing – review & editing, Conceptualization. PdM: Supervision, Writing – original draft, Writing – review & editing, Conceptualization.

## References

[1] Ma GL Murphy JD Martinez ME Sicklick JK. Epidemiology of gastrointestinal stromal tumors in the era of histology codes: results of a population-based study. Cancer Epidemiol biomark Prev Publ Am Assoc Cancer Res Cosponsored Am Soc Prev Oncol. (2015) 24:298–302. doi: 10.1158/1055-9965.EPI-14-1002 PMC4294949 25277795

[2] Sharma AK Kim TS Bauer S Sicklick JK. Gastrointestinal stromal tumor: new insights for a multimodal approach. Surg Oncol Clin N Am. (2022) 31:431–46. doi: 10.1016/j.soc.2022.03.007 PMC10185044 35715143

[3] Yamamoto H Oda Y Kawaguchi KI Nakamura N Takahira T Tamiya S. c-kit and PDGFRA mutations in extragastrointestinal stromal tumor (gastrointestinal stromal tumor of the soft tissue). Am J Surg Pathol. (2004) 28:479–88. doi: 10.1097/00000478-200404000-00007 15087667

[4] Fagkrezos D Touloumis Z Giannila M Penlidis C Papaparaskeva K Triantopoulou C. Extra-gastrointestinal stromal tumor of the omentum: a rare case report and review of the literature. Rare Tumors. (2012) 4:e44. doi: 10.4081/rt.2012.e44 23087800 PMC3475951

[5] El Charif MH Amro S Boulos F Khalife M Shamseddine A Assi H. Extra-gastrointestinal stromal tumors (EGISTs): A case report for a mischief entity. Med (Baltimore). (2023) 102:e33394. doi: 10.1097/MD.0000000000033394 PMC10063283 37000068

[6] Casali PG Blay JY Abecassis N Bajpai J Bauer S Biagini R. Gastrointestinal stromal tumours: ESMO–EURACAN–GENTURIS Clinical Practice Guidelines for diagnosis, treatment and follow-up. Ann Oncol. (2022) 33:20–33. doi: 10.1016/j.annonc.2021.09.005 34560242

[7] Wilhelm SM Dumas J Adnane L Lynch M Carter CA Schütz G. Regorafenib (BAY 73-4506): a new oral multikinase inhibitor of angiogenic, stromal and oncogenic receptor tyrosine kinases with potent preclinical antitumor activity. Int J Cancer. (2011) 129:245–55. doi: 10.1002/ijc.25864 21170960

[8] Grothey A Cutsem EV Sobrero A Siena S Falcone A Ychou M. Regorafenib monotherapy for previously treated metastatic colorectal cancer (CORRECT): an international, multicentre, randomised, placebo-controlled, phase 3 trial. Lancet. (2013) 381:303–12. doi: 10.1016/S0140-6736(12)61900-X 23177514

[9] Demetri GD Reichardt P Kang YK Blay JY Rutkowski P Gelderblom H. Efficacy and safety of regorafenib for advanced gastrointestinal stromal tumours after failure of imatinib and sunitinib (GRID): an international, multicentre, randomised, placebo-controlled, phase 3 trial. Lancet. (2013) 381:295–302. doi: 10.1016/S0140-6736(12)61857-1 23177515 PMC3819942

[10] Bruix J Qin S Merle P Granito A Huang YH Bodoky G. Regorafenib for patients with hepatocellular carcinoma who progressed on sorafenib treatment (RESORCE): a randomised, double-blind, placebo-controlled, phase 3 trial. Lancet. (2017) 389:56–66. doi: 10.1016/S0140-6736(16)32453-9 27932229

[11] Mithoowani S Laureano M Crowther MA Hillis CM. Investigation and management of erythrocytosis. CMAJ. (2020) 192:E913–8. doi: 10.1503/cmaj.191587 PMC7829024 32778603

[12] McMullin MF Harrison CN Ali S Cargo C Chen F Ewing J. A guideline for the diagnosis and management of polycythaemia vera. A Br Soc Haematol Guideline Br J Haematol. (2019) 184:176–91. doi: 10.1111/bjh.15648 30478826

[13] Arber DA Orazi A Hasserjian R Thiele J Borowitz MJ Le Beau MM. The 2016 revision to the World Health Organization classification of myeloid neoplasms and acute leukemia. Blood. 2016;127(20):2391-2405. Blood. (2016) 128:462–3. doi: 10.1182/blood-2016-06-721662 27069254

[14] Miettinen M Monihan JM Sarlomo-Rikala M Kovatich AJ Carr NJ Emory TS. Gastrointestinal stromal tumors/smooth muscle tumors (GISTs) primary in the omentum and mesentery: clinicopathologic and immunohistochemical study of 26 cases. Am J Surg Pathol. (1999) 23:1109–18. doi: 10.1097/00000478-199909000-00015 10478672

[15] Lin J Liao W Wang J Li W Tang X Li H. Primary extra-gastrointestinal stromal tumor of retroperitoneum: Clinicopathologic characteristics and prognosis of six cases. Front Oncol. (2023) 13:1033598. doi: 10.3389/fonc.2023.1033598 36895492 PMC9990817

[16] Agaimy A. Gastrointestinal stromal tumors (GIST) from risk stratification systems to the new TNM proposal: more questions than answers? A review emphasizing the need for a standardized GIST reporting. Int J Clin Exp Pathol. (2010) 3:461–71. PMC2897103 20606727

[17] Naranjo CA Busto U Sellers EM Sandor P Ruiz I Roberts EA. A method for estimating the probability of adverse drug reactions. Clin Pharmacol Ther. (1981) 30:239–45. doi: 10.1038/clpt.1981.154 7249508

[18] Rawat BPS Jagannatha A Liu F Yu H. Inferring ADR causality by predicting the Naranjo Score from Clinical Notes. AMIA Annu Symp Proc. (2021) 2020:1041–9. PMC8075501 33936480

[19] Bosbach B Deshpande S Rossi F Shieh JH Sommer G de StanChina E. Imatinib resistance and microcytic erythrocytosis in a KitV558Δ;T669I/+ gatekeeper-mutant mouse model of gastrointestinal stromal tumor. Proc Natl Acad Sci USA. (2012) 109:E2276–2283. doi: 10.1073/pnas.1115240109 PMC3427109 22652566

[20] Van Der Veldt AAM Boven E Vroling L Broxterman HJ Van Den Eertwegh AJM Haanen JG. Sunitinib-induced hemoglobin changes are related to the dosing schedule. J Clin Oncol. (2009) 27:1339–40. doi: 10.1200/JCO.2008.20.6151 19188672

[21] Alexandrescu DT McClure R Farzanmehr H Dasanu CA. Secondary erythrocytosis produced by the tyrosine kinase inhibitors sunitinib and sorafenib. J Clin Oncol. (2008) 26:4047–8. doi: 10.1200/JCO.2008.18.3525 18711201

[22] Dulgar O Cil I Zirtiloglu A Tural D. Long-lasting response with polycythemia to third-line axitinib treatment in metastatic renal cell carcinoma: Very rare case presentation. J Oncol Pharm Pract Off Publ Int Soc Oncol Pharm Pract. (2019) 25:1512–5. doi: 10.1177/1078155218790342 30058939

[23] Fanelli M Caputo F Cerma K Gelsomino F Bari A Dominici M. Pazopanib-related secondary polycythemia in metastatic myxofibrosarcoma: A case report and review of the literature. J Oncol Pharm Pract. (2021) 27:766–70. doi: 10.1177/1078155220950440 32838682

[24] Tam BY Wei K Rudge JS Hoffman J Holash J Park SK VEGF modulates erythropoiesis through regulation of adult hepatic erythropoietin synthesis. (2006) 12(7):793-800. doi: 10.1038/nm1428 16799557

[25] Wang W Cheng J Mallon C Al-Marrawi MY Holder S Joshi M. Symptomatic secondary polycythemia induced by anti-VEGF therapy for the treatment of metastatic renal cell carcinoma: A case series and review. Clin Genitourin Cancer. (2015) 13:e391–395. doi: 10.1016/j.clgc.2015.07.003 26303589

[26] Sherwood JB Shouval D. Continuous production of erythropoietin by an established human renal carcinoma cell line: development of the cell line. Proc Natl Acad Sci USA. (1986) 83:165–9. doi: 10.1073/pnas.83.1.165 PMC322812 3455754

[27] Tripathi A Jacobus S Feldman H Choueiri TK Harshman LC. Prognostic significance of increases in hemoglobin in renal cell carcinoma patients during treatment with VEGF-directed therapy. Clin Genitourin Cancer. (2017) 15:396–402. doi: 10.1016/j.clgc.2016.12.009 28040423

